# The impact of AI anchor anthropomorphism on users’ willingness to co-create value in tourism live-streaming contexts: the mediating role of social presence and the moderating role of perceived control

**DOI:** 10.3389/fpsyg.2025.1724176

**Published:** 2026-02-02

**Authors:** Qiongwei Ye, Yuting Li, Yumei Luo, Zhilin Pang

**Affiliations:** 1 Business School, Yunnan University of Finance and Economics, Kunming, Yunnan, China; 2 School of Business and Tourism Management, Yunnan University, Kunming, Yunnan, China; 3 Commercial Business Management Department, Beijing Daxing International Airport, Beijing, China

**Keywords:** AI anchors, anthropomorphism, human–machine value co-creation, perceived control, social presence, tourism live streaming

## Abstract

With the advancement of large language models and multimodal interaction technologies, AI anchors capable of substituting human hosts have been increasingly applied in the live streaming e-commerce field, demonstrating anthropomorphic characteristics that extend beyond physical appearance. Among these applications, the impact of the anthropomorphism level of AI anchors on users’ willingness to engage in human–machine value co-creation in tourism live streaming contexts remains an underexplored yet critical area. Existing studies mostly focus on the impact of anthropomorphism on purchase intention, but overlook the underlying mechanism in high-interaction contexts. Grounded in the social response theory, social presence theory and self-determination theory, this study investigates tourism live streaming as a contextual setting through experimental designs involving two levels of anthropomorphism (high vs. low). It systematically examines the impact of AI anchor anthropomorphism on users’ willingness to co-create value, the mediating role of social presence, and the moderating role of perceived control. The findings indicate that: (1) The level of anthropomorphism exhibited by AI anchors significantly and positively influences users’ willingness to participate in human–machine value co-creation, with participants in the high anthropomorphism condition reporting significantly greater willingness than those in the low anthropomorphism condition; (2) Social presence mediates this relationship; and (3) Perceived control negatively moderates the path between anthropomorphism and social presence—higher perceived control attenuates the positive effect of anthropomorphism on social presence, but does not moderate the direct relationship between anthropomorphism and willingness to co-create. This study elucidates users’ dual psychological needs for “social connection” and “autonomous control” in human–machine collaborative settings, highlights the importance of balancing these competing demands, and offers both theoretical insights and practical implications for the design of AI-driven interactions in tourism live streaming.

## 1 Introduction

With the global expansion of the digital economy, artificial intelligence (AI) has emerged as a pivotal force driving business model innovation. AI-powered virtual humans, introduced as a novel technological paradigm, have entered the e-commerce domain. Among these applications, the AI host model integrating “AI + tourism live streaming”—characterized by situational experiential value and real-time interactivity—has become a key catalyst for transforming and upgrading the e-commerce sector. Digital avatars with highly realistic appearances and behavioral traits are projected to account for over 60% of the market share in China’s virtual human industry, while the global virtual human market is expected to exceed USD 270 billion by 2030 (Thomala, 2025). As an emerging medium for human-computer interaction, AI hosts function not only as channels for disseminating tourism product information but also as enablers of technologically driven reconstructions in consumer pathways to value co-creation. In this context, understanding the mechanisms underlying user participation in value co-creation facilitated by AI hosts has become a critical research agenda for academia in the study of intelligent e-commerce and a strategic priority for industry in optimizing live streaming operations.

Value co-creation theory posits that consumers have evolved from passive recipients of services into collaborative partners who actively participate in value creation through interaction and resource integration. Unlike user engagement, which emphasises the degree of user involvement, value co-creation stresses the substantive enhancement or transformation of value achieved through shared activities (Jaakkola and Alexander, 2014). Tourism livestreaming possesses the distinctive characteristics of high interactivity and strong experiential dependency, which further reinforce the agency of users in experience construction and value generation. In tourism livestreaming scenarios, human–machine value co-creation manifests through the collaborative process where audiences, AI anchors, and digital technologies synergise to generate value, such as real-time interactions (including viewer comments, questions, and feedback) between audiences and AI anchors. Thus, user value co-creation in this study refers to users’ motivation and readiness to actively participate in tourism product value creation through interaction with AI anchors. Serving as the human–machine interface, AI anchors significantly enhance consumer emotional involvement through their professionalism, appeal, and credibility. This fosters trust and stimulates co-creation behaviors such as sharing, recommending, and purchasing, thereby elevating destination brand value (Lee et al., 2021). Unlike traditional human hosts, AI hosts exhibit dual attributes: “instrumentality,” which enables efficient information dissemination, and “human-likeness,” which fosters social interaction potential. While instrumentality enhances operational efficiency in live streaming, human-likeness plays a pivotal role in stimulating users’ willingness to engage. Therefore, incorporating “anthropomorphism” as the core independent variable is not only a necessary response to the dual nature of AI hosts but also establishes a critical analytical lens for uncovering the underlying psychological mechanisms through which users respond to realistic intelligent agents. Furthermore, the anthropomorphic design of AI anchors lays the groundwork for the co-creation of value by enhancing human-like perception. Such anthropomorphic interaction stimulates users’ sense of social presence—the perception of genuine coexistence with the anchor—thereby fostering deeper engagement (Wong and Wong, 2025). Simultaneously, users’ sense of control during interaction influences their acceptance of the anthropomorphic experience, which in turn modulates their willingness to engage in co-creation of value (Mulcahy et al., 2023).

Existing studies have confirmed that anthropomorphic design can enhance users’ sense of closeness and thereby facilitate interaction (Sun and Tang, 2024), yet it may also elicit negative perceptions due to the “uncanny valley effect” (Diel and Lewis, 2024), indicating that its influence on user behavior exhibits non-linear characteristics. However, three critical research gaps persist in the current literature: First, most studies focus on the impact of anthropomorphism on isolated psychological variables—such as trust or satisfaction—and fail to systematically elucidate the sequential psychological mechanisms through which anthropomorphism drives users’ willingness to co-create value in the highly interactive context of tourism live streaming. Second, social presence—a core construct reflecting “the sense of engaging in real social interaction”—has been empirically shown to mediate behavioral intentions in human-computer interaction (Zhang et al., 2024). Nevertheless, whether it fully mediates the effect of anthropomorphism in the context of AI host-facilitated value co-creation remains insufficiently validated. Third, value co-creation fundamentally relies on reciprocal interaction. Yet, traditional human-computer interaction research predominantly frames users’ sense of control within the “efficiency paradigm,” equating high control with high satisfaction (Xu and Ruan, 2023), while neglecting the possibility that, in the socially rich environment of tourism live streaming, perceived control may instead moderate the relationship between anthropomorphism and downstream outcomes. The absence of empirical attention to this potential boundary condition limits the applicability of existing findings and hinders the development of context-sensitive AI host design.

Building on the identified research gaps, this study focuses on the context of tourism live streaming and addresses three sequential research questions: (1) Does the level of anthropomorphism in AI hosts significantly influence consumers’ willingness to co-create value? (2) Does social presence mediate the relationship between anthropomorphism and willingness to co-create value—specifically, can anthropomorphism enhance co-creation behavior by strengthening users’ “sense of real social interaction”? (3) Does users’ need for control moderate the effect of anthropomorphism on social presence, thereby generating distinct pathways that shape willingness to co-create? To address these questions, the study, based on the social response theory, social presence theory and self-determination theory, constructs a comprehensive theorical framework. The sense of relatedness and interpersonal connection emphasized by social presence theory—attained through social interactions—are inherently consistent with the need for relatedness in self-determination theory. More importantly, as a positive psychological experience, social presence itself can simultaneously satisfy users’ needs for relatedness and competence, thereby fostering their intrinsic motivation to participate in value co-creation; in turn, self-determination theory systematically explains how these fulfilled psychological needs drive users’ behavioral intentions (Gao et al., 2018). Therefore, this study regards social presence as a key mediating mechanism linking external stimuli and intrinsic psychological motivation. This framework enables a systematic examination of the underlying psychological mechanisms through which anthropomorphism influences user engagement in value co-creation.

The theoretical, mechanistic, and practical contributions of this study are threefold. Theoretically, it transcends the “efficiency-oriented” paradigm prevalent in traditional human-computer interaction by redefining AI hosts as “value co-creation partners” rather than mere functional tools—a novel conceptualization that extends the applicability of value co-creation theory to intelligent agent-mediated contexts and offers a new framework for understanding human–machine symbiotic collaboration. Mechanistically, the study identifies and empirically validates the “control paradox”—the phenomenon whereby a high user need for control significantly attenuates the positive effect of anthropomorphism on social presence. This challenges the linear assumption that “higher anthropomorphism always leads to better outcomes,” thereby delineating a critical psychological boundary condition in reciprocal human–machine interactions. Practically, the findings underscore the necessity for AI hosts to balance instrumental attributes (efficiency in information transmission) with social attributes (capacity for emotional resonance), offering actionable strategic guidance for tourism e-commerce enterprises in designing AI-driven live streaming interactions that are both efficient and empathetic—such as through adaptive realism levels and calibrated control permissions.

## 2 Literature review and research hypotheses

### 2.1 Anthropomorphism and willingness to co-create value

In the live streaming e-commerce sector, AI digital humans demonstrate significant application value by simulating human appearance and possessing emotional interaction capabilities. They integrate voice, interaction, and emotional technologies to build core functions (Lan et al., 2023). Anthropomorphism refers to the simulation of human characteristics through virtual agents, enabling users to perceive non-living entities as having human attributes. Operationally, its impact on users manifests in three dimensions: visual, interactive, and emotional (Zhang et al., 2022).

Existing research has confirmed the extensive impact of anthropomorphism on users’ cognition, emotions, and behavior (Ayache et al., 2022), yet significant research gaps remain regarding its underlying mechanisms. Firstly, most studies focus on the visual attributes of anthropomorphism, neglecting the mediating role of users’ psychological anthropomorphic cognition. Particularly in highly interactive live-streaming scenarios, the driving pathway of anthropomorphism intensity towards value co-creation remains unclear (Liu and Zhang, 2025). Secondly, while social response theory explains users’ automatic social responses to personified entities (Nass and Moon, 2000), existing research fails to elucidate how such social perceptions translate into concrete value co-creation behaviors (Xie et al., 2023). Finally, current literature primarily examines the direct impact of anthropomorphism on single psychological variables (Akhtar et al., 2024), lacking a systematic explanation of how personification facilitates value co-creation through multiple psychological mechanisms. In particular, the unique operational mechanisms of artificial intelligence digital humans as co-creation agents require further exploration (Fouad et al., 2024).

Rooted in social response theory, individuals automatically engage in anthropomorphic cognition of non-human objects and display social interactive behaviors. Its core tenets highlight social responses as dynamic, context-dependent, and shaped by multi-dimensional interactions that modulate individual/group engagement with social influence and communication. Within human-robot interaction (HRI), humanoid robots’ appearance, voice, and responsiveness strongly impact users’ perceived social presence, humanness, and continued usage intentions. Meta-analyses validate that robots’ facial and kinetic social cues boost trust and social presence, aligning with the Computers Are Social Actors (CASA) paradigm (Xu and Ruan, 2023). In marketing, embedding artificial empathy in AI agents enhances customers’ affective and social experiences by narrowing the human-AI interaction gap, though effectiveness is context-dependent (Thompkins et al., 2022). Chatbot response time research indicates that user experience moderates social responses, with delays exerting distinct effects on novice versus experienced users (Gnewuch et al., 2022). Furthermore, robot-initiated social touch alleviates stress and strengthens perceived intimacy in human-robot relationships, underscoring nonverbal cues’ role in robotic social responses (Williams and Aaker, 2002). Collectively, this offers a fundamental psychological basis for explaining why users form emotional connections and co-creation intentions with AI anchors. Consequently, this study employs this theory to clarify the underlying mechanisms—at the level of cognitive automaticity—through which anthropomorphism influences users’ social presence and value co-creation intentions. In value co-creation scenarios, anthropomorphism stimulates users’ willingness to collaborate by reinforcing their social perception and emotional connection with artificial intelligence. Specifically, when AI anchors are endowed with more human-like traits, they evoke stronger social presence and emotional bonds among users, making them more inclined to trust and interact with them. This enhanced trust and perceived social presence narrows the psychological distance between users and AI, rendering interactions more authentic. Consequently, it motivates users to actively participate in co-creation activities, such as providing feedback, sharing ideas, or collaborating to complete service experiences (Mulcahy et al., 2023).

From a mechanism perspective, anthropomorphic design prompts users to perceive AI as “social actors,” thereby activating their inherent social interaction norms and collaborative tendencies (Akhtar et al., 2024). Empirical research indicates that anthropomorphic cues at both linguistic and visual levels enhance users’ subjective well-being and likelihood of participating in co-creation. Moreover, heightened levels of anthropomorphism cultivate intimacy and self-consistency, further motivating users to integrate AI into their activities and contribute to value co-creation (Alabed et al., 2022). For instance, Xie et al. (2023) found that anthropomorphic cues enhance users’ trust and closeness towards AI, thereby increasing their willingness to interact. Shen et al. (2024) further indicated that highly anthropomorphized AI alleviates users’ perceived identity threat, creating psychological safety conditions conducive to co-creation behavior. Therefore, based on multiple mechanisms triggered by anthropomorphism—including social cognition, emotional bonding, and reduced psychological distance—this study proposes the following hypotheses:

*H1*: The anthropomorphism level of the AI anchor has a positive impact on consumers’ willingness to engage in human–machine value co-creation with the AI anchor.

### 2.2 Mediating role of social presence

Social presence refers to the psychological experience through which users perceive “the presence of others” in a media environment, and it has been widely recognized as a key mechanism influencing user behavior in virtual interaction and live streaming contexts (Chen and Liao, 2022). It is not merely a technological attribute but is also shaped by social practices, individual traits, contextual factors, and the construction of participants’ shared social identity. Multiple theoretical perspectives—including social information processing, construal level theory, and telepresence—explain variations in social presence across communication modes and psychological distance (Oh et al., 2018).

In AI-driven interaction contexts, the anthropomorphism of digital humans may significantly enhance users’ social presence: highly anthropomorphic virtual avatars can more effectively transmit emotional signals and improve interaction authenticity, thereby strengthening users’ “sense of co-presence” (Gao et al., 2023; Wang et al., 2023). For instance, in a study on live streaming e-commerce, Gao et al. (2023) found that the anthropomorphic characteristics of virtual hosts indirectly increased consumers’ purchase intention by enhancing social presence, which aligns with the aforementioned logic regarding how the anthropomorphism of AI digital humans influences user behavior.

Furthermore, social presence plays a critical mediating role between anthropomorphism level and behavioral willingness. Gao et al. (2023) noted that social presence significantly mediated the impact of interactivity on purchase intention in live streaming interactions; similarly, in the context of AI digital humans, anthropomorphic design can promote users’ willingness to co-create value by enhancing social presence. A study by Huang et al. (2022) also indicated that immersive experience—one form of social presence—exerted a full mediating role between social presence and purchase intention. This suggests that anthropomorphism is not merely a superficial simulation of appearance or behavior; rather, it deeply influences users’ willingness to participate and behavioral tendencies by stimulating social presence. However, existing research still has limitations: most studies focus on e-commerce or entertainment live streaming contexts, and there remains a lack of research on the mediating mechanism of social presence in the context of human–machine value co-creation involving and AI anchor (Bai and Tan, 2024).

Based on the above theoretical analysis and combined with the aforementioned framework of how the anthropomorphism of AI anchor influences value co-creation, this study proposes the following hypothesis:

*H2*: Social presence mediates the impact of the anthropomorphism level of AI Anchor on the willingness to engage in human–machine value co-creation.

### 2.3 Moderating role of perceived control

Grounded in self-determination theory, human behavior is driven by three basic psychological needs: autonomy, competence, and relatedness. It also highlights the social and environmental conditions that support or thwart these psychological needs, influencing motivation, development, and wellness (Deci and Ryan, 2008). Organizational behavior research shows that different types of motivation within SDT predict employee well-being, attitudes, and performance, with intrinsic motivation and identified regulation being particularly important for positive work outcomes (den Van Broeck et al., 2016). Among these, the core of the autonomy need is an individual’s desire to control their own behavior (Deci and Ryan, 1985). This need was first conceptualized as “perceived control,” specifically referring to the way individuals adjust their responses when facing challenges, and essentially representing the degree of confidence in managing and controlling their own emotions. Thus, the self-determination theory provides a crucial theoretical anchor for understanding the moderating role of “perceived control” in this study, helping to clarify the central position of this fundamental psychological need in driving human–machine interaction behavior. By integrating this theory with anthropomorphism research, we can explain from a motivational perspective why a high need for perceived control inhibits users’ processing of social cues. This enables the construction of a complete theoretical moderation pathway linking anthropomorphism, perceived control, and social presence.

In tourism livestreaming scenarios, co-creation manifests as viewers engaging in collaborative processes of experience construction and content generation through real-time interaction, whilst perceived user control directly determines their engagement depth and co-creative willingness. When livestream settings offer interactive features such as real-time chat and content selection, heightened perceived control not only enhances users’ autonomy and immersion but also serves as a pivotal psychological mechanism for stimulating co-creative behaviors like contributing feedback and sharing ideas (Liu and Zhang, 2024). Combined with the aforementioned mediating mechanism of social presence, as a key variable satisfying the autonomy need, the moderating role of perceived control between the anthropomorphism of AI digital humans and social presence urgently requires exploration.

In this study, perceived control within tourism livestreaming contexts is defined as the perception of autonomy and mastery that viewers experience over the livestream content and their overall experience during real-time interaction, information perception, and engagement in decision-making processes (Wang and Guo, 2024). Specifically, when AI anchors possess highly anthropomorphic traits, users automatically activate cognitive schemata associated with interpersonal interaction. However, users with higher perceived control typically demand greater autonomy over the interaction process, making them more sensitive to the AI anchor’s autonomy and directionality and prone to psychological resistance. They perceive such interactions as encroaching on their decision-making autonomy, which undermines the intended enhancement of social presence through anthropomorphic design (Hassan et al., 2022; Hu et al., 2024). Mechanistically, users with high perceived control, who exhibit strong autonomous motivation, demonstrate lower reliance on social cues from AI. Consequently, anthropomorphic features have a relatively limited effect on enhancing their sense of social presence. Conversely, users with low perceived control are more inclined to seek social cues and emotional support from AI, in which case anthropomorphic design proves significantly more effective in enhancing their sense of social presence (Munnukka et al., 2022).

Integrating theoretical deduction and empirical research, and combined with the aforementioned positive impact of the anthropomorphism of AI on social presence, this study proposes the following hypothesis regarding the moderating effect:

*H3*: Perceived control negatively moderates the impact of the anthropomorphism of AI Anchor on social presence. That is, for consumers with a high level of perceived control, the positive promoting effect of the anthropomorphism level of AI anchor on social presence is weaker.

**Figure 1:**
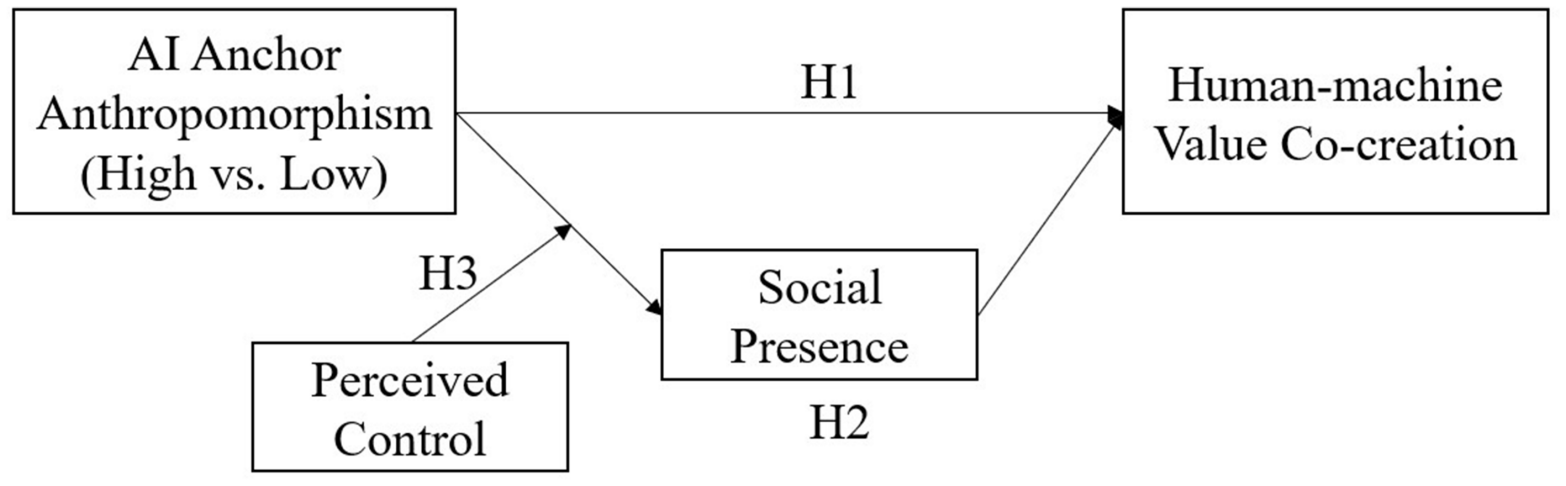
Research model.

Figure 1 shows the research model of this study.

## 3 Research methodology

### 3.1 Research design

This study adopted a combination of scenario-based experiments and questionnaires for data collection. Specifically, the scenario-based experiment method enables direct manipulation of the anthropomorphism level of virtual humans in a relatively controlled environment, thereby better examining the impact of different anthropomorphism levels on the dependent variable. To test Hypotheses H1–H3, this study conducted three experiments. Experiment 1, as a foundational study, adopted a single-factor design to preliminarily validate the significant positive impact of AI anchors’ anthropomorphism levels on users’ value co-creation willingness (H1). Building on this, Experiment 2 introduced social presence as a mediating variable, uncovering the underlying mechanism through which anthropomorphism operates (H2). Experiment 3 further incorporated perceived control as a moderator, delineating the boundary conditions of the path from anthropomorphism to social presence (H3). Collectively, these three experiments form a comprehensive evidence chain covering main effects, mediation mechanisms, and moderating effects, thereby enhancing the rigour and depth of the research conclusions.

All constructs were measured using mature scales revised to adapt to the research scenario (see Table 1), with a unified 5-point Likert scale (1 = Strongly Disagree, 5 = Strongly Agree) adopted, and the specific measurement basis is as follows: willingness to engage in human–machine value co-creation, referring to the research by Shamim et al. (2017), includes 6 items to measure the intensity of participants’ willingness to engage in tourism service co-creation with AI hosts; anthropomorphism of AI AI anchor, using the scale developed by Ruijten et al. (2019) and Shen et al. (2024), consists of 6 items to assess participants’ perception of the anthropomorphism level of AI hosts; for control variables, given that AI AI anchor belong to the category of new products (a factor that may influence users’ willingness to co-create), this study referred to the research by Roehrich (2004) and set 4 items to measure consumers’ innovation preference, in addition to demographic variables (including gender, age, educational background, and occupation type); meanwhile, following the suggestion by Lu et al. (2021), screening items such as “difficulty in scenario imagination” and “system authenticity” were added to identify and exclude invalid experimental data.

**Table 1:** Measurement of constructs.

Constructs	Items	Source
Human–machine value co-creation	I enjoy interacting in this AI anchor’s live streaming room.	Shamim et al. (2017)
	I am willing to ask this AI anchor for detailed information about tourism products or services.	
	I am willing to share my own tourism knowledge or experiences with this AI anchor.	
	When this AI anchor asks for my suggestions on improving tourism routes or services, I am willing to respond actively.	
	When this AI anchor invites me to participate in co-design, I am willing to participate actively.	
	I am willing to provide my feedback on the innovative tourism services recommended by this AI anchor.	
AI anchor anthropomorphism	This AI anchor’s speech and behavior feel very natural to me.	Ruijten et al. (2019), Shen et al. (2024)
	This AI anchor feels friendly and approachable to me.	
	This AI anchor’s performance is as energetic as a real person.	
	I think this AI anchor has human-like thinking ability.	
	This AI anchor can provide targeted suggestions based on my questions.	
	This AI anchor can understand my emotions and needs.	
Social presence	During the live interaction, the “presence” of this AI anchor feels very distinct to me.	Chen et al. (2023), Liao et al. (2024)
	When interacting with this AI anchor, I feel that we are co-present in the current space.	
	Throughout the interaction, I can sense a shared atmosphere between us.	
	I pay attention to this AI anchor’s responses during the interaction.	
	Communicating with this AI anchor makes me feel like I am interacting with a real social partner.	
Psychological discomfort and sense of insecurity	When the appearance of the AI anchor is overly realistic, I feel uneasy or uncomfortable.	Wang et al. (2023), Williams and Aaker (2002)
	The expressions or movements of the AI anchor make me feel “uncanny” or “unnatural.”	
	I fear that the AI anchor may collect or misuse my personal information.	
	When interacting with the AI anchor, I doubt the true intentions behind it.	
Perceived control	When interacting with this AI anchor, I feel the entire interaction process is under my control.	Pedroso-Chaparro et al. (2023)
	I believe I can freely have the interactions I want with this AI anchor.	
	When interacting with this AI anchor, I feel I can determine the direction of the interaction.	
	This AI anchor makes me feel that I am leading this interaction, rather than being led by it.	
Innovation preference	I enjoy trying new technologies or services (such as AI anchors and virtual reality live streaming).	Roehrich (2004)
	I am usually the first among my friends to experience new technologies.	
	When I hear about new digital services launching, I take the initiative to learn about them.	
	I believe using new technologies enhances my life/consumption experience.	

### 3.2 Experiment 1

Experiment 1 adopted a single-factor, two-level (high anthropomorphism vs. low anthropomorphism) group design to examine the impact of AI digital human anthropomorphism on the willingness to engage in human–machine value co-creation (Hypothesis H1). A pre-experiment was conducted before the formal experiment to verify the control effect of the experimental materials.

#### 3.2.1 Pre-experiment measurement

The pre-experiment used the video scenario priming method: participants were asked to watch a tourism live streaming video featuring an AI anchor to immerse themselves in the experimental scenario, and then completed the questionnaire administration. The specific design is as follows:

For the selection of experimental background materials, combining previous literature and actual live streaming experience, an AI digital human anchor group-buying live streaming scenario themed “7-day In-depth Tour of Shangri-La, Yunnan” was constructed. The core of the video presented the real-time interaction between the AI anchor and the audience, including the process of jointly discussing and designing tourism services or products, so as to simulate a real value co-creation scenario.

**Figure 2:**
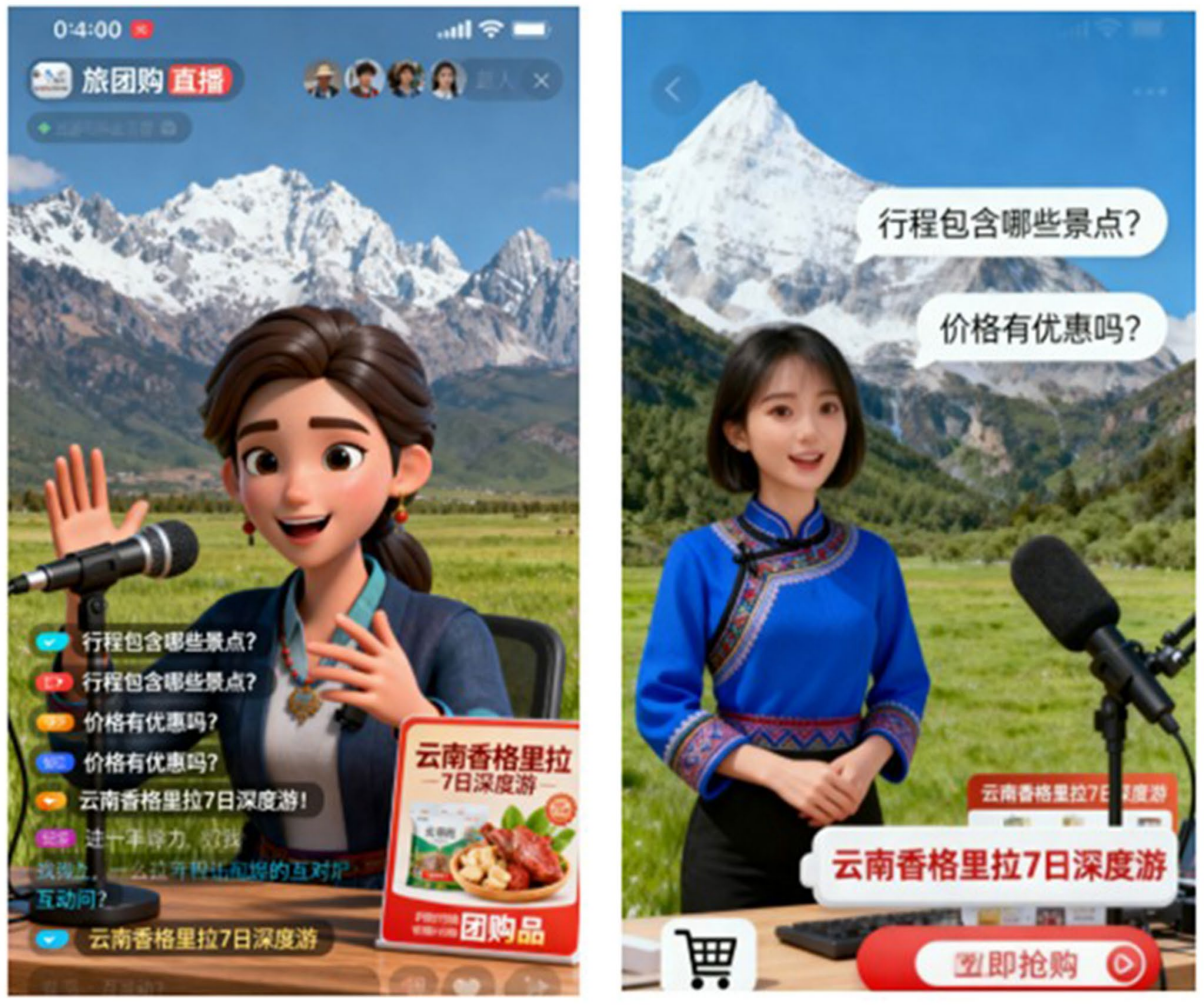
Screenshots of the manipulation video for AI anchor live streaming tourism group-buying service in Experiment 1.

After watching the video, participants were required to fill out a questionnaire based on their experience. The experiment was divided into two experimental groups (high anthropomorphism and low anthropomorphism), with the differences between the groups as follows:

High anthropomorphism group: The AI digital human adopted a hyper-realistic 3D image, with appearance, expressions, and movements consistent with real humans; its voice was recorded by a real person, with natural intonation and emotional fluctuations; during interaction, it would actively smile and nod in response to audience questions, use personalized language (e.g., “We can decide whether to add a hiking route tomorrow based on bullet screen votes!”), and dynamically adjust its answers according to audience questions (as shown in the right panel of Figure 2).

Low anthropomorphism group: The AI digital human adopted a simplified cartoon 3D image, which was a geometric model without facial details; its movements showed mechanical characteristics, with fixed cyclic gestures and no facial expression changes; its voice was a synthetic electronic voice, without emotional fluctuations and no modal particles at the end of sentences; it could only recognize keywords and reply with preset answers (as shown in the left panel of Figure 2).

The recruited participants were randomly divided into the high anthropomorphism group and the low anthropomorphism group, with 34 participants in each group. The scenario settings and the measurement logic of each variable were completely consistent between the two groups; the only difference was the anthropomorphism level of the AI anchor (corresponding to the high and low anthropomorphism video designs mentioned above). The pre-experiment was conducted in April 2025, and a total of 68 valid questionnaires were collected (34 from each group).

**Figure 3:**
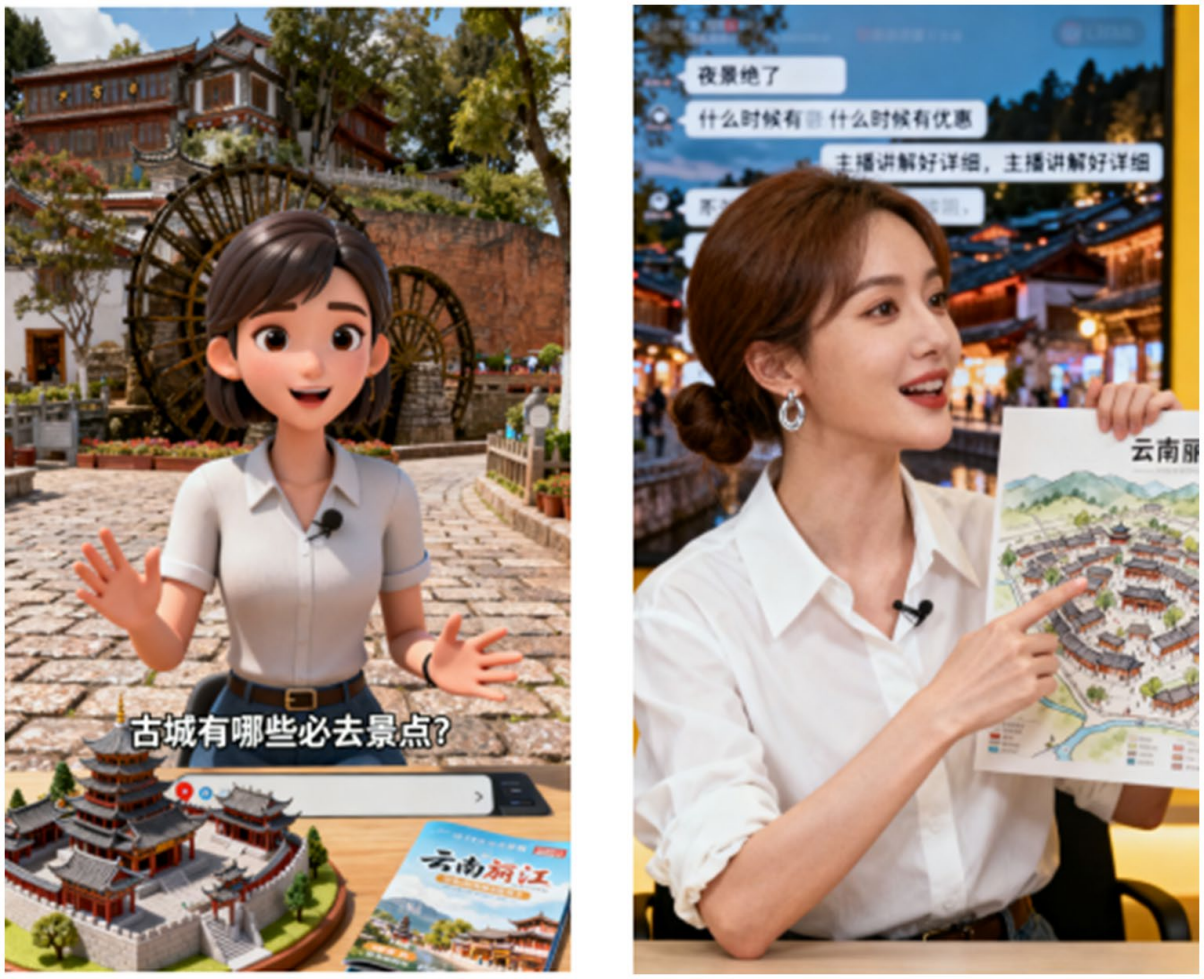
Screenshots of the AI anchor live streaming video for tourism services in Experiment 3.

Scenario authenticity test: The scores of the 68 participants on the authenticity of the experimental scenario showed that the average scores of the three items—"the scenario is easy to understand” (*M* = 4.24), “can clearly imagine the scenario I am in” (*M* = 3.84), and “can fully immerse myself in the scenario” (*M* = 3.78)—were all at a high level, indicating that the experimental scenario design is reasonable and realistic (see Figure 3).

Independent variable manipulation validity test: The results of the independent samples t-test showed that the mean score of anthropomorphism perception in the high anthropomorphism group was significantly higher than that in the low anthropomorphism group (M_high anthropomorphism = 4.05, M_low anthropomorphism = 2.31, *t* = −10.456, *p* < 0.01). This proves that the experimental materials effectively manipulated the independent variable of “AI anchor anthropomorphism,” laying the foundation for the formal experiment.

#### 3.2.2 Experimental manipulation and procedure

The design of the formal experiment was consistent with that of the pre-experiment. A total of 328 participants were recruited via Wenjuanxing (an online questionnaire platform). After screening and excluding 36 invalid questionnaires (due to incorrect filling, missing filling, or failure to immerse in the experimental scenario), 292 valid samples were finally obtained, with an effective rate of 89%. Among the samples, there were 93 males and 199 females; most participants were aged 18–55 years (accounting for 93%); their educational backgrounds were mainly junior college (27%) and undergraduate (59%); and approximately 77% of the participants had previous experience with digital human services. This study selected a relatively homogeneous sample of college students, which could effectively control exogenous variables such as age and education level, and more clearly reveal the intrinsic causal relationship between variables (Wysocki et al., 2022). Meanwhile, females accounted for 68% of the sample, which was consistent with the gender structure of mainstream users in fields such as tourism live streaming and experiential consumption, ensuring the phenomenological representativeness of the sample (Wan and Lu, 2024). The results of the independent samples t-test showed that there was a significant difference in anthropomorphism level between the high anthropomorphism group and the low anthropomorphism group (M_high anthropomorphism = 3.64, M_low anthropomorphism = 2.153, t = −13.832, *p* < 0.01) (see Table 2), indicating that the independent variable manipulation was successful. In addition, the potential influences of consumers’ innovation preference (Cronbach’s *α* = 0.889) and demographic characteristics were controlled in the statistical analysis.

**Table 2:** Summary of key results from Experiment 1.

Constructs	High anthropomorphism group (*n* = 146)	Low anthropomorphism group (*n* = 146)	Group comparison
Anthropomorphism	3.644 (0.825)	2.153 (1.008)	*t* = −13.832, *p* < 0 0.001
Human–machine value co-creation	3.704 (0.765)	2.255 (1.021)	*F* (1, 290) = 188.398, *p* < 0.001

Values in parentheses denote standard deviation.

#### 3.2.3 Result analysis and discussion

A one-way ANOVA was further used to explore the impact of AI digital human anthropomorphism level (high vs. low) on users’ willingness to co-create value. Table 2 showed that the users’ willingness to co-create value in the high anthropomorphism group was significantly higher than that in the low anthropomorphism group (M_high anthropomorphism = 3.70, M_low anthropomorphism = 2.26, *F* (1, 290) = 188.398, *p* < 0.001). This indicates that the anthropomorphism level effectively promotes consumers’ willingness to engage in human–machine value co-creation, thus verifying Hypothesis H1.

### 3.3 Experiment 2

The materials and procedures of Experiment 2 were basically consistent with those of Experiment 1, with the addition of measuring social presence—the experiment aimed to re-test Hypothesis H1 and examine the mediating role of social presence in the relationship between variables. The measurement of social presence adopted the scale developed by Chen et al. (2023) and Liao et al. (2024), which included 5 measurement items. To ensure data validity, the requirements for participants and data screening criteria were the same as those in Experiment 1. After reading the experimental materials, participants were asked to imagine themselves being in the aforementioned service scenario and fill out a questionnaire (covering the manipulated variable, willingness to engage in human–machine value co-creation, social presence, and other control variables) based on their true thoughts.

In addition, considering that an excessively high level of anthropomorphism may trigger the uncanny valley effect and disrupt the path effect of willingness to co-create value, participants’ psychological discomfort and sense of insecurity caused by the digital human in the materials were also measured. The former referred to the scale by Williams and Aaker (2002), while the latter referred to the scale by Wang et al. (2023) (see Table 1).

#### 3.3.1 Experimental manipulation and procedure

A total of 380 participants were recruited via Wenjuanxing (an online questionnaire platform) for Experiment 2. After excluding 40 pieces of invalid data, 340 valid samples were obtained, with an effective rate of 89%. Among the valid samples, there were 112 males and 228 females; educational backgrounds were mainly undergraduate (63%) and postgraduate or above (22%); and ages were mainly concentrated between 18 and 40 years old, accounting for approximately 96%. Different from Experiment 1, the proportion of students was relatively high (about 83%).

The independent samples t-test showed a significant difference in anthropomorphism evaluation scores between the two experimental groups (M_high anthropomorphism = 3.44, M_low anthropomorphism = 2.38, t = −11.499, *p* < 0.01), indicating that the experimental manipulation was effective. Anthropomorphism (Cronbach’s *α* = 0.854), social presence (Cronbach’s α = 0.876), and willingness to engage in human–machine value co-creation (Cronbach’s α = 0.836) all showed good internal consistency. Meanwhile, the average scores of psychological insecurity and discomfort were 3.42 and 3.84, respectively, suggesting that the anthropomorphic materials used in this experiment may not cause significant psychological insecurity or discomfort. In addition, the potential influences of consumers’ innovation preference and demographic characteristics were also controlled in the statistical analysis.

#### 3.3.2 Result analysis and discussion

Consistent with Experiment 1, there was a significant difference in the willingness to engage in human–machine value co-creation between the two experimental groups (M_high anthropomorphism = 3.98, M_low anthropomorphism = 3.55, *F*(1, 338) = 41.501, p < 0.01). Further one-way ANOVA results showed a significant difference in social presence between the two groups (M_high anthropomorphism = 3.50, M_low anthropomorphism = 2.58, F(1, 338) = 95.227, p < 0.01). This indicates that highly anthropomorphic AI digital humans are indeed more effective in enhancing users’ social presence than lowly anthropomorphic ones.

To examine the mediating role of social presence in the relationship between anthropomorphism and willingness to co-create value, we conducted three regression analyses following the mediation effect testing procedure proposed by Shrout et al. (2002). The results are detailed in Table 3. First, results of Model 0 indicate that anthropomorphism exerts a significant positive influence on willingness to co-create value (*β* = 0.497, *p* < 0.001). Secondly, Model 1 results indicate that anthropomorphism also exerts a significant positive influence on social presence (*β* = 0.846, p < 0.001). Finally, in Model 2, when both anthropomorphism and social presence were included in the regression equation, the effect of social presence on the willingness to co-create value was significant (*β* = 0.251, p < 0.001). Concurrently, while the direct effect of anthropomorphism on the willingness to co-create value remained significant, its coefficient value showed a marked decrease relative to the total effect.

**Table 3:** Experiment 2: Analysis of the mediating effect of social presence (*N* = 340).

Predictor Variables	Human–machine value co-creation	Social presence	Human–machine value co-creation
	Model 0	Model 1	Model 2
Intercept	2.000^***^ (0.296)	1.384^***^ (0.338)	1.537^***^ (0.278)
Control variables			
Gender	−0.348*** (0.087)	−0.130 (0.092)	−0.315*** (0.084)
Age	−0.030 (0.090)	0.102 (0.095)	−0.055 (0.087)
Education	−0.088 (0.054)	−0.041 (0.058)	−0.078 (0.052)
Occupation	0.000 (0.069)	−0.047 (0.074)	0.011 (0.067)
Innovation preference	0.471*** (0.056)	0.513*** (0.059)	0.342*** (0.060)
Independent variable			
AI anchor anthropomorphism	0.497*** (0.081)	0.846*** (0.086)	0.284** (0.089)
Social presence	--	--	0.251*** (0.050)
*R* ^2^	0.290	0.367	0.340
Adjusted *R*^2^	0.277	0.355	0.326
Δ*R*^2^	0.080	0.183	0.130
*F*	22.636	32.128	24.445

Values in parentheses denote standard errors. *p < 0.05, **p < 0.01, ***p < 0.001. “--” indicates that the variable was not included in the regression model.

The aforementioned findings collectively indicate that social presence partially mediates the relationship between AI anchor anthropomorphic and the willingness to co-create value. To further strengthen the robustness of this conclusion, we employed Bootstrap sampling (with 5,000 repeated samples) to test the indirect effect. As shown in Table 4, the confidence interval of the mediating path of social presence (*β*_indirect effect = 0.212, LLCI = 0.112, ULCI = 0.325) did not include 0—indicating that social presence plays a significant mediating role between anthropomorphism level and willingness to co-create value with AI digital humans, thus supporting Hypothesis H2. The direct effect between anthropomorphism and willingness to engage in human–machine value co-creation was also significant (*β*_direct effect = 0.284, LLCI = 0.109, ULCI = 0.460, CI did not include 0), which proves that social presence has a partial mediating effect and further verifies Hypothesis H1.

**Table 4:** Results of bootstrap test for mediating effects.

Effect type	Mediator	Effect size	Standard error (SE)	*t*-value	*p*-value	95% confidence interval LLCI ULCI	
Direct effect	–	0.284	0.089	3.194	0.002	0.109	0.460
Indirect effect	Social Presence	0.212	0.0542	–	–	0.112	0.325

### 3.4 Experiment 3

The procedure of Experiment 3 was basically consistent with the previous two experiments, with the addition of measuring perceived control.

#### 3.4.1 Pre-experiment measurement

To simulate a real value co-creation scenario, this study referred to the operational practice of “Caiyun”—the first digital cultural tourism promotion officer of Yunnan (a major tourism province in China)—and designed a live streaming video of an AI digital human anchor promoting tourism products of “Lijiang Ancient Town, Yunnan” as the experimental material (see Figure 3). Participants were first asked to watch the video, which simulated the live streaming scenario of a tourism platform and presented the process of the AI anchor interacting with the audience to jointly design tourism products or services. Subsequently, participants filled out a questionnaire based on the scenario of the video they watched.

The manipulation of anthropomorphism level was consistent with Experiments 1 and 2 in its core dimensions, but adapted the specific content to the Lijiang Ancient Town scenario. The key distinctions between the high and low anthropomorphism conditions are summarized in Table 5. The detailed textual scripts used for the scenario priming are provided in Appendix A for reference.

**Table 5:** Key differences in anthropomorphism manipulation for Experiment 3.

Dimension	High anthropomorphism condition	Low anthropomorphism condition
Appearance and voice	Hyper-realistic 3D model; natural, emotional human voice	Simplified cartoon 3D model; synthetic, monotone electronic voice
Language style	Warm, engaging, and conversational (e.g., “I’ll take you to explore,” “Leave your wishes.”)	Formal, mechanical, and instructional (e.g., “Important notice,” “Operation: type.”)
Interaction and responsiveness	Dynamic, proactive, and socially rich; uses open-ended questions to simulate mutual decision-making	Static, reactive, and functionally oriented; relies on fixed commands and preset Q&A
Overall perception goal	To be perceived as a sociable partner	To be perceived as an instrumental tool

On this basis, the experiment applied corresponding video intervention stimuli to participants in the two experimental groups (based on previous work), and then asked them to answer relevant questions. The questionnaire design was the same as that in Experiment 2, with four measurement items for the “perceived control” variable added, referring to the scale developed by Pedroso-Chaparro et al. (2023) (see Table 1). This study conceptualizes perceived control as a user’s psychological state within a specific interactive context. Participants assessed their perceived level of control during that specific simulated interaction immediately after watching the assigned livestreaming video, using the provided scale. A total of 78 participants were randomly divided into the two experimental groups. The independent samples t-test showed a significant difference in anthropomorphism evaluation between the two groups (M_high anthropomorphism = 3.46, M_low anthropomorphism = 2.01, *t* = −9.817, *p* < 0.01), indicating that the experimental manipulation was effective and the materials could effectively induce participants’ anthropomorphism evaluation.

#### 3.4.2 Experimental manipulation and procedure

A total of 432 participants took part in the formal experiment. After data verification and screening, 398 valid questionnaires were obtained, including 144 males and 254 females. Approximately 96% of the participants were aged between 18 and 40 years old. Regarding educational backgrounds: 6.3% had senior high school education or below, 8.8% had junior college education, 63.8% had undergraduate education, and 21.1% had postgraduate education or above. The four variables—anthropomorphism (Cronbach’s *α* = 0.866), willingness to co-create value (Cronbach’s α = 0.847), social presence (Cronbach’s α = 0.885), and perceived control (Cronbach’s α = 0.766)—all showed good reliability. In addition, the potential influences of consumers’ innovation preference and demographic characteristics were also controlled in the statistical analysis.

#### 3.4.3 Result analysis and discussion

One-way ANOVA results showed a significant difference in the willingness to engage in human–machine value co-creation between the two groups (M_high anthropomorphism = 3.41, M_low anthropomorphism = 3.04, *F* (1, 396) = 17.125, *p* < 0.01), which was basically consistent with the previous two experiments. This study further tested Hypothesis H3 using the stepwise regression method (see Table 6).

**Table 6:** Experimental 3: analysis results of the moderated mediated model (*N* = 398).

Predictor variables	Social presence			Human–machine value co-creation	
	Model 0	Model 1	Model 2	Model 3	Model 4
Intercept	1.015*** (0.264)	0.704** (0.252)	0.213 (0.307)	1.577*** (0.232)	1.348*** (0.219)
Control variables					
Gender	−0.244* (0.094)	−0.201* (0.089)	−0.168* (0.089)	−0.414*** (0.081)	−0.349*** (0.077)
Age	0.065 (0.104)	0.105 (0.098)	0.099 (0.097)	0.037 (0.090)	0.003 (0.084)
Education	−0.110 (0.061)	−0.023 (0.057)	−0.010 (0.057)	−0.113 (0.053)	−0.106* (0.049)
Occupation	0.056 (0.059)	0.060 (0.056)	0.059 (0.055)	0.002 (0.051)	−0.021 (0.048)
Innovation preference	0.600*** (0.058)	0.592*** (0.055)	0.539*** (0.065)	0.553*** (0.051)	0.360*** (0.054)
Independent variable					
AI anchor anthropomorphism		0.596*** (0.084)	1.425*** (0.341)	0.340*** (0.077)	0.146* (0.077)
Mediator variable					
Social presence			--		0.326*** (0.043)
Moderator variable					
Perceived control			0.200* (0.080)		--
Interaction term					
AI anchor anthropomorphism × perceived control			−0.261* (0.104)		--
*R* ^2^	0.223	0.312	0.325	0.303	0.391
Adjusted *R*^2^	0.213	0.301	0.311	0.292	0.380
Δ*R*^2^	0.223	0.089	0.013	0.035	--
*F*	22.473	29.484	23.403	28.345	35.800

Values in parentheses denote standard errors. *p < 0.05, **p < 0.01, ***p < 0.001. “--” indicates that the variable was not included in the regression model.

Experiment 3 aimed to examine the moderating role of perceived control. We employed the PROCESS macro to analyze the data. Results are presented in Table 6. First, Model 0 only included control variables. On this basis, the independent variable “AI anchor anthropomorphism “was added to form Model 1. The results showed that AI anchor anthropomorphism had a significant positive effect on social presence (*β* = 0.596, *p* < 0.001). The results of Model 2 revealed that the interaction term between anthropomorphism and perceived control significantly and negatively predicted social presence (*β* = −0.261, *p* = 0.012). This indicates that perceived control plays a significant moderating role in the process whereby anthropomorphism influences social presence, thus supporting Hypothesis H3.

**Figure 4:**
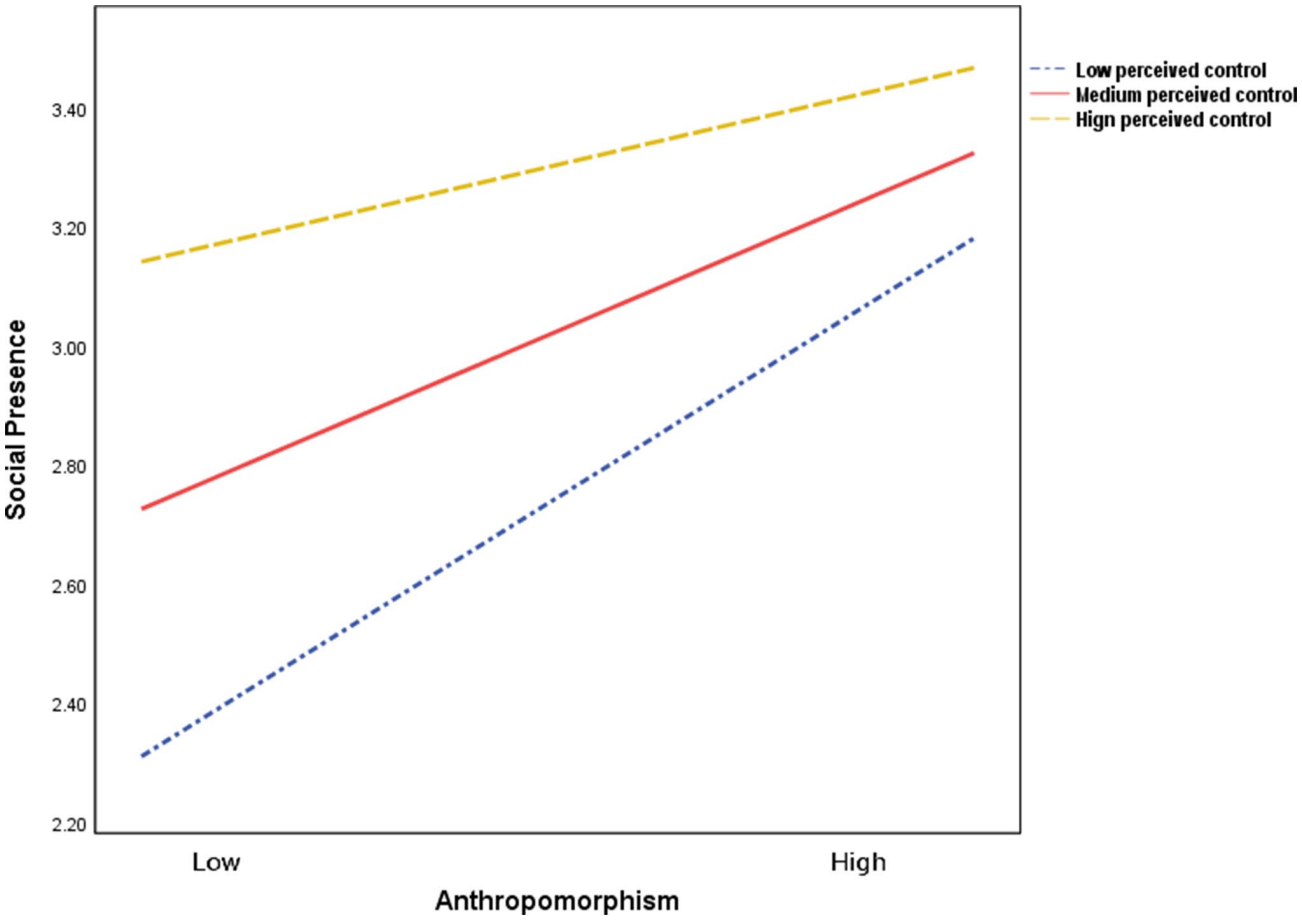
Analysis of the moderating role of perceived control.

To further explore the specific role of the moderating variable, simple slope analysis was conducted. After mean-centering the moderating variable, the differences in impact effects under different levels were observed by adding or subtracting one standard deviation (Dalal and Zickar, 2011). As can be intuitively seen in Figure 4: when perceived control was low, the anthropomorphism level exerted the strongest positive impact on social presence (*β* = 0.871, *t* = 6.831, *p* < 0.05); when perceived control was moderate, the impact of anthropomorphism remained significant but was weaker than that under low perceived control (*β* = 0.598, *t* = 6.655, *p* < 0.05); when perceived control was high, the impact of anthropomorphism was the weakest yet still significant (*β* = 0.326, *t* = 2.559, *p* < 0.05). The specific conditional effect analysis is shown in Table 7. In general, the higher the perceived control, the weaker the positive promoting effect of anthropomorphism level on social presence, indicating that the direction of the moderating effect is “negative buffering”—thus supporting Hypothesis H3.

**Table 7:** Analysis of conditional indirect effects.

Perceived control level	Effect value	Boot standard error	Boot LLCI	Boot ULCI
Low level (−1 SD)	0.2632	0.0545	0.1603	0.3734
Mean level	0.1944	0.0383	0.1217	0.2720
High level (+1 SD)	0.1255	0.0448	0.0423	0.2189
Index of moderated mediation	−0.0850	0.0395	−0.1639	−0.0097

The results of Model 3 in Table 6 also verify Hypothesis H1, i.e., anthropomorphism exerts a significant positive effect on the willingness to co-create value (Model 3, *β* = 0.340, *p* < 0.001). Additionally, the impact of social presence on the willingness to engage in human–machine value co-creation remains a significant positive effect (Model 4, *β* = 0.326, *p* < 0.001). When combined with the results of Model 1, Hypothesis H2 is supported.

Finally, and most crucially, we examined the moderated mediation effect. Analysis of the conditional indirect effects revealed that under low, medium, and high levels of perceived control, the indirect effect values mediated through social presence were 0.263, 0.194, and 0.126,respectively. Their Bootstrap confidence intervals all excluded zero, indicating that the indirect effect was significant across all levels. More significantly, the moderated mediation index was −0.085, with its 95% Bootstrap confidence interval [−0.164, −0.010] excluding zero. This provides conclusive statistical evidence for the core hypothesis: “Perceived control negatively moderates the indirect path through which personification design influences co-creation willingness via social presence.” In summary, the findings from Experiment Three fully validate our theoretical model: social presence serves as the key mediating mechanism through which personification design drives users’ willingness to co-create value, and the efficacy of this mechanism is constrained by users’ perceived control. When users perceive higher levels of control, the strength of this mediating pathway is systematically reduced.

Synthesizing the findings of the three aforementioned studies, the research hypotheses of Experiment 1 and Experiment 2 are all verified, and the first hypothesis of Experiment 3 is also valid.

## 4 Discussion and conclusions

This study transcends the narrow focus on “whether anthropomorphism is effective” and systematically investigates the more nuanced question of “for whom and under what conditions it exerts its influence.” By establishing perceived control as a key boundary condition, it clarifies the dual roles of AI anchors in value co-creation and validates the theoretical hypotheses through multiple experimental contexts. The core findings are as follows:

(1) A significant positive relationship exists between the level of anthropomorphism in AI digital humans and consumers’ willingness to co-create value: higher anthropomorphism is associated with greater user engagement in co-creation;
(2) Social presence partially mediates the relationship between anthropomorphism level and willingness to co-create value, indicating that anthropomorphism indirectly promotes co-creation behavior by enhancing users’ “sense of real social interaction.”;
(3) Perceived control exerts a negative moderating effect on the anthropomorphism–social presence path: high perceived control attenuates the positive influence of anthropomorphism on social presence.

### 4.1 Theoretical contributions

First, by introducing the “Control Paradox,” this study challenges the linear cognitive framework regarding the effects of anthropomorphism and provides a new explanatory perspective for the “uncanny valley effect.” Existing literature generally holds that higher anthropomorphism leads to more positive user responses by enhancing social presence. However, our research finds that this positive effect is systematically constrained by users’ perceived control. When users have high perceived control over the interaction process, the social presence evoked by highly anthropomorphic AI anchors is instead weakened. This finding stands in stark contrast to the traditional efficiency paradigm, which posits that high control is equivalent to high satisfaction. Our results indicate that in tourism live streaming contexts rich in social cues, high perceived control may make users more sensitive to the autonomy and guidance of AI, perceiving it as a threat or intrusion to their decision-making autonomy, thereby generating psychological reactance and offsetting the social benefits brought by anthropomorphism. This provides a potential new mechanism for understanding the “uncanny valley effect”: users’ discomfort may stem not only from visual realism and eeriness but also, in certain interactive contexts, from the psychological threat of “loss of control” triggered by anthropomorphic AI. Future research can directly test this mechanism.

Second, this study clarifies the unique role of AI anchors in value co-creation, extending their conceptualization from functional tools to social co-creation partners. Previous research has mostly regarded AI as a tool for improving efficiency or simply analogized its anthropomorphic effects to those of human anchors. Our findings reveal that AI anchors possess dual instrumental and social attributes simultaneously, and indicate that users’ willingness to co-create stems from two complementary rather than competitive pathways: one based on the sense of control over the interaction process, and the other on the sense of social connection with AI. This conclusion echoes the research calls in human-computer interaction and social robotics, emphasizing that the value of AI anchors lies not only in their technical capabilities but also in their ability to facilitate meaningful social interactions. This transcends the mindset of merely “replicating human behaviors,” pointing toward a new paradigm of human–machine collaboration integrating efficiency and emotion, and providing a more refined theoretical framework for understanding the role of intelligent agents in value co-creation.

Finally, this study extends the research context of AI anchors from transactional e-commerce to experiential tourism live streaming, verifying and deepening Social Presence Theory and Self-Determination Theory in high-involvement service scenarios. In high-involvement contexts such as tourism live streaming—where information depth, experiential immersion, and decision-making autonomy are emphasized—our research confirms that social presence serves as the core psychological mechanism driving user participation, while perceived control acts as a key boundary condition. This responds to the consensus in existing literature regarding the transformative role of AI in service- and experience-driven industries, and for the first time, provides an integrated theoretical framework and empirical support for the interaction between social presence and perceived control in the co-creation context of tourism live streaming. This work advances AI interaction research toward greater contextual sensitivity and analytical precision, laying a theoretical foundation for innovative marketing and consumer engagement strategies based on the co-creation of information and experiential values in the tourism industry.

### 4.2 Practical implications

Building on the core behavioral pathway of “Design–Perception–Behavior” (anthropomorphic design → social presence → willingness to co-create value), this study proposes a three-tiered strategic framework for the operational management and interaction design of AI anchors in tourism live streaming:

First, at the strategic design level: Shift AI anchor development from an “instrumental” to a “relational” paradigm and enrich the conceptual depth of anthropomorphism. Enterprises should move beyond superficial realism—such as appearance and voice—and instead focus on constructing AI’s “personality script” and “contextual intelligence” by embedding stable personality traits (e.g., affability, professionalism) and empathetic capabilities. This enables the AI to recognize emotions and needs in user comments and engage in two-way social interactions that transcend mere information delivery—such as proactively responding to personalized requests—thereby establishing a robust social foundation for value co-creation.

Second, at the experience operation level: Prioritize the enhancement of social presence to cultivate an immersive social environment. Given its mediating role, platforms should design interaction mechanisms centered on “co-presence.” On one hand, establish standardized interaction rituals—such as AI-initiated greetings and real-time responses to chat messages; on the other, create explicit co-creation opportunities—like allowing users to vote on itinerary adjustments during the live stream. These practices transform users from passive audiences into active co-creators, strengthening their sense of participation and belonging.

Third, at the risk management level: Strategically balance users’ perceived control to preserve the integrity of the immersive social experience. In light of the negative moderating effect of perceived control, enterprises should avoid offering complex or unnecessary control features and instead adopt the principle of “strong guidance, weak control”—providing limited, well-timed choices at key decision points (e.g., selecting tourism product packages). This approach satisfies users’ fundamental need for autonomy while preventing excessive control from fragmenting attention and undermining the social immersion, thus achieving an optimal equilibrium between anthropomorphic engagement and user agency. This recommendation aligns with the consensus within immersive technology research that achieving optimal experiences requires balancing presence with user agency, whilst avoiding increased user burden or diminished immersion resulting from poorly designed control functionalities.

### 4.3 Limitations and future research

First, current research on anthropomorphic behavior largely adopts a holistic perspective, neglecting individual-level differences. In fact, anthropomorphism theory encompasses multiple psychological and cognitive levels; focusing on a single level risks overlooking the dynamic interplay between these dimensions. For example, when users perceive high visual similarity but experience weak emotional resonance, they may react negatively due to the disconfirmation of expectations—the perceived gap between anticipated and actual social responsiveness.

Second, the sample is predominantly composed of young college students with high educational attainment. Future studies should broaden the participant pool to include individuals across a wider range of ages and professional backgrounds to enhance the generalizability of the findings.

Third, the sample exhibits an imbalanced gender distribution. Subsequent research could conduct targeted investigations focusing specifically on male users to explore potential gender-based variations in responses to anthropomorphic AI.

## Data Availability

The original contributions presented in the study are included in the article/supplementary material, further inquiries can be directed to the corresponding author.

## Appendix

### Appendix A: Detailed Scenario Scripts for Experiment 3 Manipulation

A1 High Anthropomorphism Condition Script Example.

High anthropomorphism group example content: “Tonight at 8 o’clock, I’ll take you to explore hidden travel experiences in Lijiang! Leave your travel wishes in the comments—3 people will be selected to win free accommodation! Want to know how to travel around Lijiang with 500 yuan? Type ‘1’ in the comments, and I’ll reveal the answer during the live stream! How many stone bridges are there in the ancient town? Answer correctly to get a 50-yuan voucher! Do you prefer a ‘cultural in-depth tour’ or an ‘internet-famous check-in route’?” For the high perceived control subgroup, the AI anchor in the video frequently provided clear options (e.g., “Next, do you want to learn about Scenic Spot A or Scenic Spot B?”) and emphasized “It’s up to you to decide.”

A2 Low Anthropomorphism Condition Script Example.

Low anthropomorphism group content: “Important notice: Hidden travel experiences in Lijiang will be unlocked during the live stream at 20:00 tonight. Everyone, enter your travel wishes in the comment section—3 people will be selected to receive free accommodation. Regarding costs: the goal is to travel around Lijiang with 500 yuan; operation: type the number ‘1’ in the comment section, and the answer will be revealed during the live stream. Do you want to know the number of existing stone bridges in Lijiang Ancient Town? Operation: type the answer in the bullet screen; condition: answer correctly to trigger a 50-yuan voucher reward.”
